# Integrated analysis of necroptosis-related lncRNAs for prognosis and immunotherapy of patients with pancreatic adenocarcinoma

**DOI:** 10.3389/fgene.2022.940794

**Published:** 2022-08-16

**Authors:** Jiantao Mo, Zhiwei Cui, Qiqi Wang, Weifan Zhang, Jie Li, Shuai Wu, Weikun Qian, Cancan Zhou, Qingyong Ma, Zheng Wang, Zheng Wu

**Affiliations:** ^1^ Department of Hepatobiliary Surgery, First Affiliated Hospital of Xi’an Jiaotong University, Xi’an, China; ^2^ Department of Obstetrics and Gynecology, First Affiliated Hospital of Xi’an Jiaotong University, Xi’an, China

**Keywords:** pancreatic adenocarcinoma, necroptosis-related lncRNAs, risk model, prognostic biomarkers, immunotherapy

## Abstract

Accumulating studies have revealed that necroptosis plays a vital role in the occurrence and development of pancreatic adenocarcinoma (PAAD). We aimed to construct a prognostic model for PAAD on the basis of necroptosis-related lncRNAs (NRLs). A coexpression network between necroptosis-related mRNAs and NRLs based on The Cancer Genome Atlas (TCGA) was constructed. Then, differentially expressed necroptosis-related lncRNAs (DENRLs) were screened from TCGA and Genotype-Tissue Expression project (GTEx) datasets. Univariate Cox regression (uni-Cox) analysis was performed on these DENRLs to identify lncRNAs significantly correlated with prognosis. Least absolute shrinkage and selection operator (LASSO) regression was performed for preventing overfitting on these lncRNAs. Multivariate Cox analysis (multi-Cox) was performed to establish a risk model based on lncRNAs that served as an independent prognostic factor. Next, the Kaplan–Meier analysis, time-dependent receiver operating characteristics (ROC), uni-Cox, multi-Cox regression, nomogram, and calibration curves were constructed to support the accuracy of the model. Gene set enrichment analysis (GSEA) and single-sample GSEA (ssGSEA) were also performed on risk groups, and it was found that the low-risk group was closely correlated with immune infiltration and immunotherapy. To further evaluate the immune differences between different clusters, we divided the patients into two clusters. Cluster 2 was more significantly infiltrated with immune cells and had higher immune scores. These results shed new light on the pathogenesis of PAAD based on NRLs and develop a prognostic model for diagnosing and guiding personalized immunotherapy of PAAD patients.

## Introduction

Pancreatic adenocarcinoma (PAAD) is the fourth leading cause of cancer-related deaths worldwide, with 5-year survival rates of less than 11% (Siegel et al., 2022). Although significant progress has been made in the treatment of pancreatic cancer in the past few decades, the postoperative prognosis of pancreatic cancer patients is not ideal because of the insidious onset and high recurrence and metastasis rate of pancreatic cancer (Neoptolemos et al., 2018; Mizrahi et al., 2020). Compared with a single clinical marker, a model integrating multiple markers can more accurately predict patient prognosis and provide targets for therapy on the basis of the development of large-scale genome sequencing technologies. Therefore, it is important to construct a molecular model that can predict patient prognosis and guide individualized therapy.

Necroptosis is a form of programmed cell death that can serve as a mode for cells to overcome apoptotic resistance (Gong et al., 2019), which plays an important role in inflammation, microbial infection, and tumor metastasis (Seifert et al., 2016; Place et al., 2021; Liu Z. et al., 2021; Yan et al., 2022). Long noncoding RNAs (lncRNAs), which are defined as RNA molecules more than 200 nucleotides in length and have limited protein-coding ability, play an important part in the development and progression of different cancers, including PAAD (Liu et al., 2019; Zhou et al., 2020). Accumulating evidence revealed that lncRNAs are involved in the occurrence and development of necroptosis (Wang et al., 2020; Harari-Steinfeld et al., 2021). However, the role of prognosis and potential molecular mechanism associated with NRLs is currently unclear in PAAD.

In the present study, we established a prognostic model based on NRLs and found that it was able to divide patients into two groups with significant differences in prognosis and immune infiltration, which may provide diverse insights into a clinical therapy for PAAD patients.

## Materials and methods

### Acquisition of datasets and patients’ information of PAAD

The PAAD RNA-seq dataset and GTEx data were downloaded from UCSC Xena (http://xena.ucsc.edu/) (including 178 tumor samples and 171 normal samples). Corresponding clinical data (including age, gender, survival time, survival status, grade, and pathological stage) were also obtained from UCSC Xena. Then we converted the data of GTEx in the form of log2 (X+0.001) to log2 (X+1) to be consistent with the data of PAAD by Strawberry Perl. The study excluded patients with missing survival information to reduce statistical bias in this analysis.

### Identification of DENRLs

A total of 67 necroptosis-related genes (NRGs) were collected from previous literature (Zhao et al., 2021). Then, 356 necroptosis-related lncRNAs (NRLs) were obtained from a correlation analysis performed between NRGs and lncRNAs of PAAD (|Pearson correlation coefficients| > 0.7, and *p* < 0.001). The mRNA–lncRNA coexpression network was visualized by the “igraph” R package. The Wilcox test was performed to explore the expression of 356 NRLs between PAAD and normal tissues with the “limma” package (Ritchie et al., 2015) in R (Rx64 4.1.2, https://www.rstudio.com/) (Log2 fold change (FC) > 1, false discovery rate (FDR) < 0.05). Finally, 172 DENRLs were obtained and visualized by the “pheatmap” R package.

### Construction and validation of a prognostic NRL model for PAAD

DENRLs’ expression data merged with the survival information of 176 patients were retrieved and regrouped into the train, test, and entire group randomly by the Perl script and the “caret” R package with a ratio of 1:1. Based on uni-Cox analysis with *p* < 0.01 as the threshold, prognosis-related lncRNAs were screened and incorporated into a Lasso regression analysis to conduct multivariate Cox proportional hazard regression analysis with *p* < 0.05 as the threshold. After narrowing the range of prognosis-related lncRNAs and preventing overfitting, a model was constructed by using the R package “glmnet” (Friedman et al., 2010; Simon et al., 2011). The risk score is calculated by the following formula:

$$\text{risk}\;\text{score}=\overset{n}{\underset{i=1}{\sum }}\text{coefficient}\left({\text{lncRNA}}^{i}\right)*\text{expression}\left({\text{lncRNA}}^{i}\right).$$

The patients were divided into low-risk and high-risk groups by the median risk score. The Chi-square test was used to evaluate the correlation of clinical factors with the model and visualized by the R package “survminer.” Univariate Cox and multivariate Cox regression analyses were developed to determine whether the risk score and clinical characteristics were independent predictors. The R package “survivalROC” was used to evaluate the accuracy of the constructed model. The larger the AUC (area under the ROC curve), the higher the prediction accuracy of the model.

### Construction and validation of the nomogram

To improve the predictive accuracy of NRLs in the model of PAAD patients, a nomogram was established for forecasting 1-, 2-, and 3-year OS integrating risk scores, age, gender, grade, T stage, N stage, and pathological stage by the “rms” R package. Then, the calibration curve was used to illustrate the discrimination between the nomogram-predicted value and the true value.

### Gene set enrichment analysis between high- and low-risk groups

We used gene set enrichment analysis (GSEA) software 4.2.1 (http://www.gsea-msigdb.org/gsea/index.jsp) to investigate the significantly predefined biological pathways between the high- and low-risk groups with the threshold: *p* < 0.01 and FDR <0.1. c2. cp.kegg.v7.1. symbols.gmt was chosen from the Molecular Signatures Database (MSigDB http://software.broadinstitute.org/gsea/msigdb/index.jsp) as the reference file. All these results were visualized by ggplot2, grid, and gridExtra R packages.

### Exploration of immune infiltration and immune checkpoints

To figure out the correlation between NRL signatures in the model and the tumor immune microenvironment, we calculated the infiltration values for PAAD patients based on seven algorithms: XCELL (Aran et al., 2017), TIMER (Li et al., 2017), QUANTISEQ (Finotello et al., 2019), MCPCOUNTER (Becht et al., 2016), EPIC (Racle et al., 2017), CIBERSORT−ABS (Newman et al., 2015), and CIBERSORT (Newman et al., 2015). Then, Spearman correlation analysis visualized in a bubble chart was used to evaluate the relationship between the immune and risk scores. Subsequently, single-sample GSEA (ssGSEA) was performed for investigating the difference between immune cells and immune function between high- and low-risk groups. The stromal score, immune score, and ESTIMATE score of each patient in different risk groups were calculated. The differences were visualized by the “ggpubr” R package. In addition, different expressions of immune checkpoint genes between the risk groups were evaluated by the “ggpubr” R package.

### Clusters based on the expression of lncRNAs in the model

As the literature illustrated previously, different subtypes usually contributed to different immune microenvironments (DeBerardinis, 2020). To explore the immunotherapy response of different subtypes, subgroups based on the expression of lncRNAs in the model by ConsensusClusterPlus (CC) (Wilkerson and Hayes, 2010) R package were performed to investigate the response to immunotherapy within them. Parameters of the consensus clustering model were set as maxK = 9, reps = 50, pItem = 0.8, pFeature = 1, clusterAlg = "km,” and distance = "euclidean.” Principal component analysis (PCA), T-distributed stochastic neighbor embedding (t-SNE), and Kaplan–Meier survival curve were constructed by the “Rtsne” R package.

### Cell culture and quantitative real-time RT-PCR (RT-qPCR)

Normal human pancreatic ductal epithelial cells hTERT-HPNE and two pancreatic cancer cells MiaPaCa-2 and BxPC-3 were purchased from the Cell Bank of Type Culture Collection of the Chinese Academy of Sciences (Shanghai, China). HPNE and MiaPaCa-2 were cultured in Dulbecco’s Modified Eagle Medium (Gibco, United States) with 10% fetal bovine serum (FBS) and 1% penicillin/streptomycin. BxPC-3 was cultured in Roswell Park Memorial Institute (RPMI) medium (Gibco, United States) with 10% FBS. A humidified atmosphere containing 5% CO_2_ at 37 °C was provided to culture all cells. A Fastgen 200 RNA isolation kit (Shanghai, China) was used to extract the total RNA according to the protocol provided. Then, the total RNA was reverse transcribed into cDNA by using the Prime Script RT reagent kit (TaKaRa, Dalian, China). RT-qPCR was performed using the CFX Manager 2.1 fluorescent quantitative PCR kit (Bio-Rad Laboratories, United States). The primer sequences were as follows: linc01089 (human), sense 5′-CCT​CTA​GCA​GAG​TGC​CTT​GG-3′ and antisense 5′-AGG​TAA​CCG​GGG​TCA​GAT​CA-3’; GAPDH (human), sense 5′-GGA​GCG​AGA​TCC​CTC​CAA​AAT-3′ and antisense 5′-GGC​TGT​TGT​CAT​ACT​TCT​CAT​GG-3’. GAPDH was used as an internal control.

## Results

### Identification of necroptosis-related lncRNAs

**FIGURE 1:**
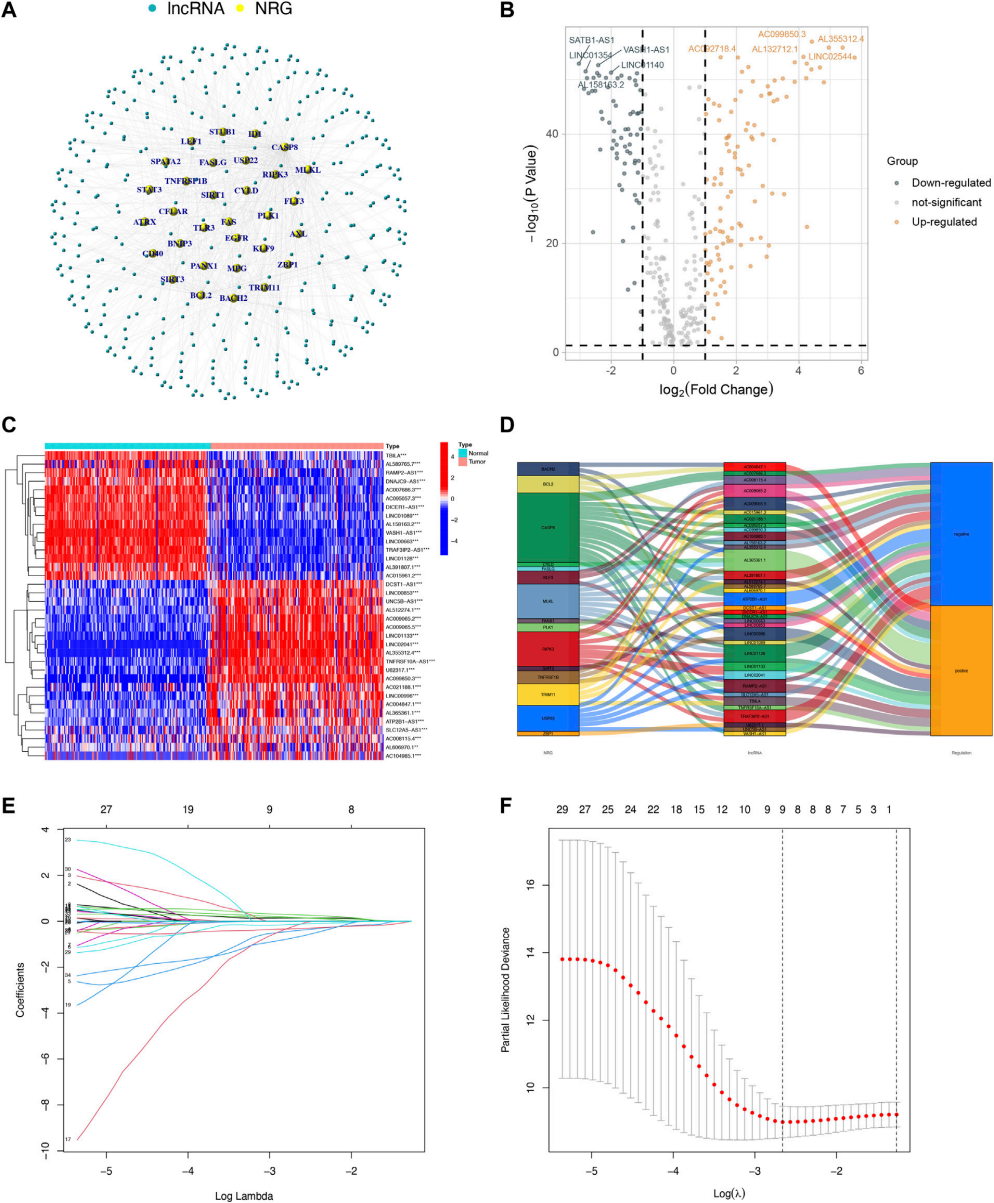
Identification and selection of NRL prognostic signatures in PAAD. **(A)** Coexpression network between NRGs and NRLs (|Pearson correlation coefficients| > 0.7, and *p* < 0.001). **(B)** Volcano plot of 172 DENRLs. **(C)** The expression profiles of 36 prognostic-related NRLs. **(D)** The Sankey diagram of prognostic NRLs, NRGs, and risk type. **(E)** The LASSO coefficient profile of necroptosis-related lncRNAs. **(F)** The 10-fold cross-validation for variable selection in the LASSO model.

A total of 178 tumor samples and 171 normal samples (167 samples from GTEx) were obtained from UCSC Xena based on TCGA and GTEx. Then we identified 356 NRLs and expression data by Pearson correlation analysis based on 69 NRGs (|Pearson correlation coefficients| > 0.7, and *p* < 0.001). The network between NRGs and NRLs are shown in Figure 1A. Subsequently, 172 DENRLs were screened between tumor and normal samples, of which 66 were downregulated and 106 were upregulated (Log2 fold change (FC) > 1, false discovery rate (FDR) < 0.05, Figure 1B).

### Construction and validation of the prognostic model based on TCGA cohorts

**FIGURE 2:**
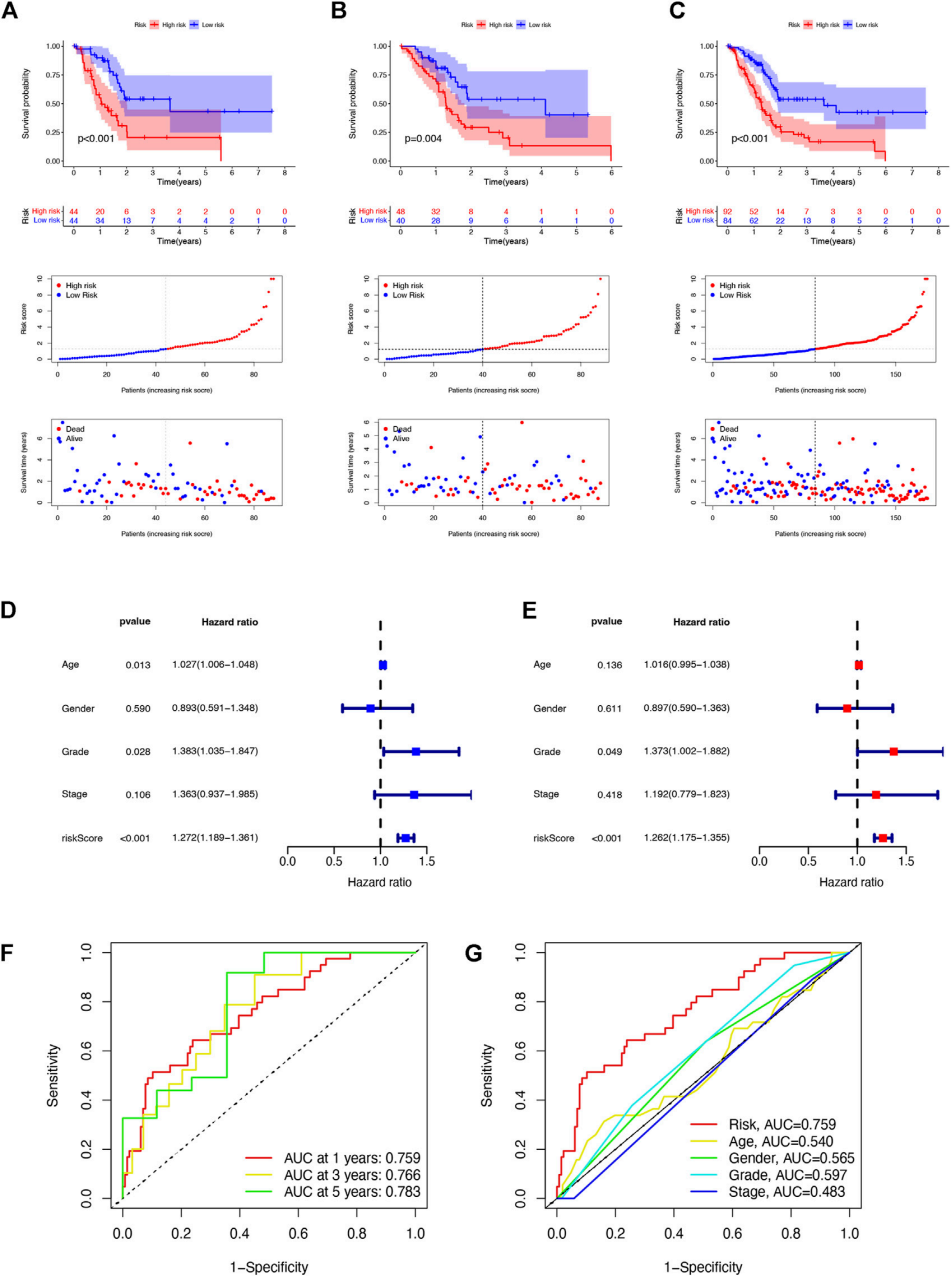
Prognosis value of the five NRL models in the train, test, and entire sets. **(A)** Risk score distribution, scatter plots, and KM survival curve analysis of patients with PAAD in the training cohort. **(B)** Risk score distribution, scatter plots, and KM survival curve analysis of patients with PAAD in the testing cohort. **(C)** Risk score distribution, scatter plots, and KM survival curve analysis of patients with PAAD in the entire cohort. **(D)** Univariate Cox regression showing that the age, grade, and risk score were associated with the OS (*p* < 0.05). **(E)** Multivariate Cox regression showing that the risk score (*p* < 0.001) was an independent prognostic indicator of the OS in patients with PAAD. **(F)** 1-, 3-, and 5-year ROC curves of the entire set. **(G)** 1-year ROC curves of the risk score and clinical characteristics.

**FIGURE 3:**
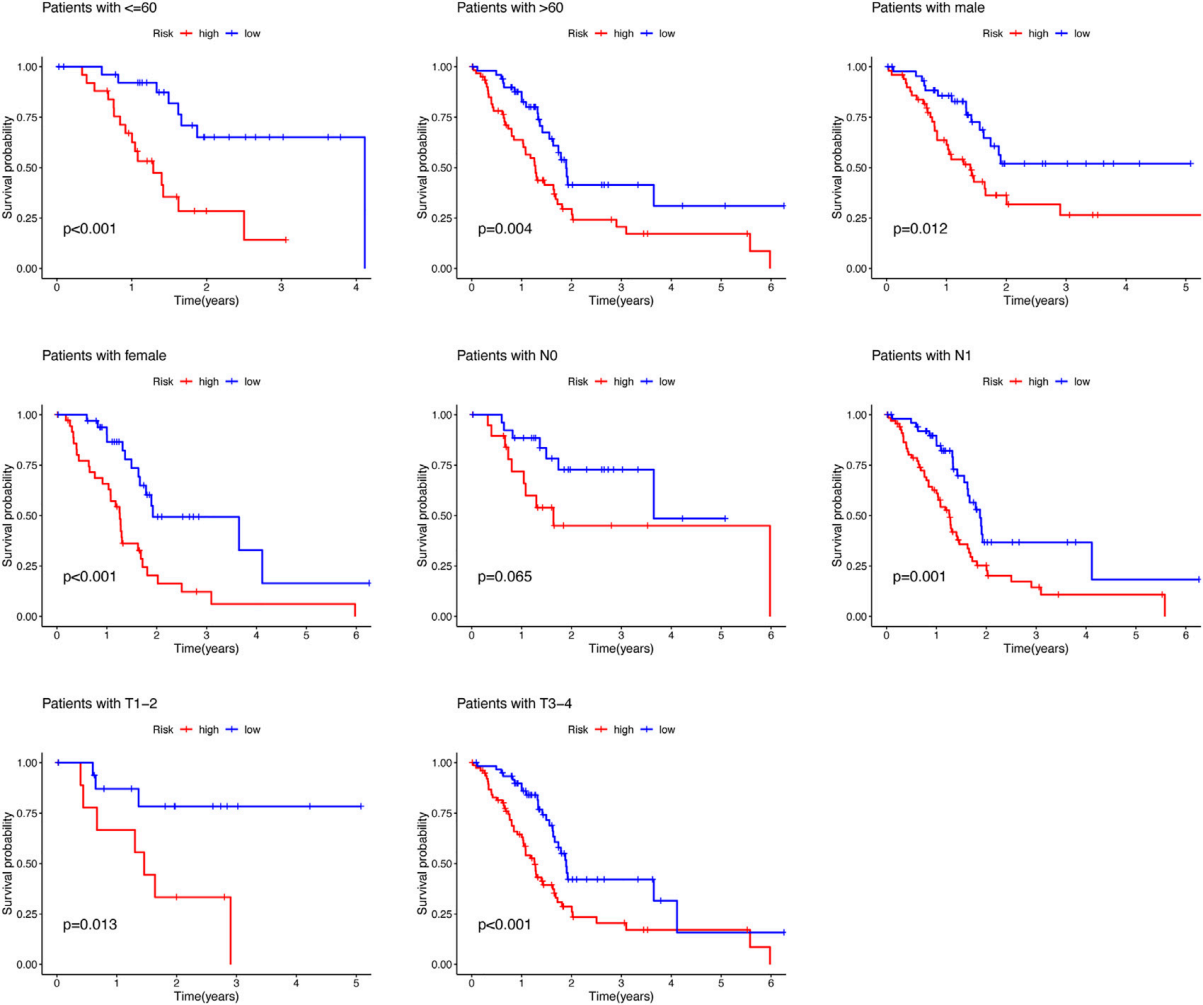
Survival rate of PAAD patients with high- and low-risk patients in the subgroups based on clinicopathological characteristics.

To predict the prognosis and provide therapeutic targets for PAAD patients, we constructed a prognostic model based on NRLs. A total of 36 prognostic-related NRLs were screened by univariate Cox regression (*p* < 0.01) and visualized with a heatmap based on their expression between tumor and normal samples (Figure 1C). The correlations between prognostic NRLs, NRGs, and the risk type were displayed by a Sankey diagram (Figure 1D). To narrow the range of prognosis-related lncRNAs and avoid overfitting, we performed Lasso regression and extracted nine NRLs (Figures 1E,F). Then, multivariate Cox proportional hazard regression analysis was conducted and five NRLs were screened to establish a prognostic NRL signature model (U62317.1, AC099850.3, LINC01089, LINC01133, and LINC00996). The risk score formula was as follows: (0.4675 × U62317.1 expression) + (0.3947 × AC099850.3 expression) + (−0.5810 × LINC01089 expression) + (0.2759 × LINC01133 expression) + (−2.0985 × LINC00996 expression). With this formula, we calculated the risk score for each patient based on personalized NRL expression levels in train, test, and entire groups and grouped them into low- and high-risk groups by the median risk score. All these results implied a significant statistical difference in the OS between the high- and low-risk groups, indicating that these developed signatures effectively predict prognosis (Figures 2A–C, Supplementary Figure S1). Subsequently, univariate and multivariate Cox regression analyses were performed to determine whether risk scores and clinical characteristics are independent prognostic factors for the OS in patients with PAAD. The hazard ratio (HR) (95% CI) for the risk score was 1.272 (1.189–1.361) in univariate Cox regression analysis (*p* < 0.001, Figure 2D) and 1.262 (1.175–1.355) in multivariate Cox regression analysis (*p* < 0.001, Figure 2E), which indicates that the NRLs in our model are independent prognostic indicators. In addition, the model was evaluated by ROC analysis at 1, 3, and 5 years to verify its predictive value, and the corresponding AUC was 0.759, 0.766, and 0.783, respectively. (Figure 2F). Furthermore, we combined the clinical information of age, gender, grade, and stage to construct a time-dependent ROC curve to predict the 1-year survival rate of patients with PAAD, and the AUC was 0.540, 0.565, 0.597, and 0.483 (Figure 2G). To evaluate the importance of NRLs in the progression of PAAD, we investigated the correlation between the risk score and clinical characteristics. As shown in Figure 3, the OS rate in the high-risk group of subgroups separated by age, gender, T stage, N stage, M stage, clinical stage, and grade was lower than that of the low-risk group (*p* < 0.05). Taken together, the risk model we constructed was feasible and reliable in identifying the risk of PAAD patients.

### Construction and verification of the nomogram

**FIGURE 4:**
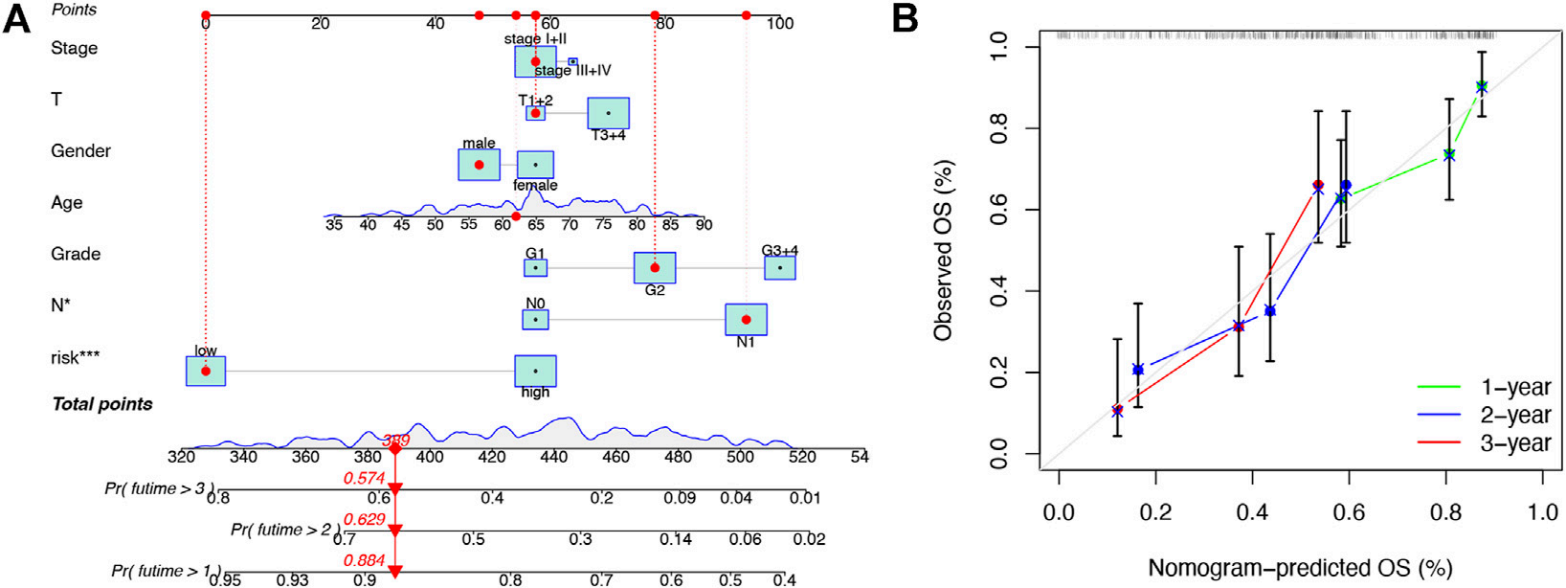
Construction and validation of the nomogram. **(A)** Prognostic nomogram that integrated risk score, age, gender, grade, T, N, and tumor stage predicted the probability of 1-, 2-, and 3-year OS. **(B)** Calibration curves for 1-, 2-, and 3-year OS.

To further enhance the predictive power of the risk model, we constructed a nomogram integrated with clinicopathological factors to predict 1-, 2-, and 3-year OS (Figure 4A). Based on the nomogram score, the 1-, 2-, and 3-year OS could be well predicted. In addition, as shown in Figure 4B, good consistency between the nomogram-predicted OS and observed OS could be found at 1, 2, and 3 years with calibration curves. The aforementioned results indicated that the nomogram had a good predictive value in PAAD patients.

### Gene set enrichment analysis between the high- and low-risk groups

**FIGURE 5:**
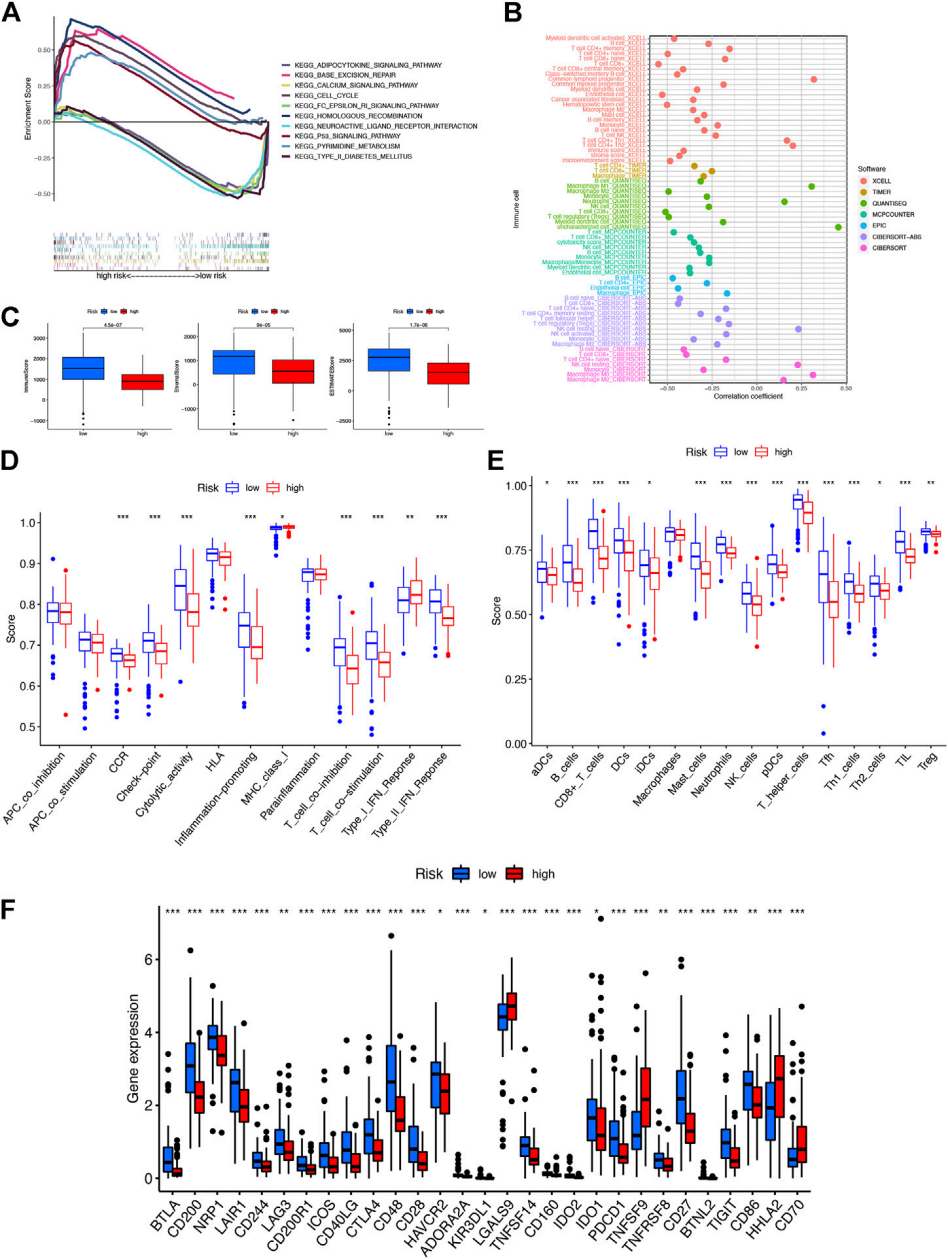
Investigation of tumor immune factors. **(A)** Top five pathways significantly enriched in the high- and low-risk groups were exhibited by GSEA. **(B)** Immune cell bubble of risk groups. **(C)** Comparison of immune-related scores between low- and high-risk groups. **(D)** Difference of immune functions in risk groups. **(E)** Difference of immune cells in risk groups. **(F)** Differential expression of 29 checkpoints’ expression in risk groups.

To further investigate the biological functions of the prognostic signature, we performed GSEA between the risk groups in the Kyoto Encyclopedia of Genes and Genomes (KEGG) pathway in the entire set (Figure 5A). The P53 signaling pathway (NES = 1.95, *p* = 0.000), cell cycle (NES = 1.91, *p* = 0.006), base excision repair (NES = 1.90, *p* = 0.000), pyrimidine metabolism (NES = 1.86, *p* = 0.000), and homologous recombination (NES = 1.85, *p* = 0.002) were enriched in the high-risk group. Neuroactive ligand-receptor interactions (NES = -1.78, *p* = 0.002), adipocytokine signaling pathway (NES = -1.75, *p* = 0.004), FC epsilon RI signaling pathway (NES = -1.75, *p* = 0.008), calcium signaling pathway (NES = -1.74, *p* = 0.008), and type II diabetes mellitus (NES = -1.70, *p* = 0.006) were enriched in PAAD samples with the low-risk group. These results indicated that the prognostic signature in the model may be associated with tumor progression and perineural invasion.

### Investigation of the correlation of immune infiltration in risk groups

A stronger correlation existed between the various immune cells and the low-risk group on different platforms (XCELL, TIMER, QUANTISEQ, MCPCOUNTER, EPIC, CIBERSORT-ABS, and CIBERSORT) exhibited with an immune cell bubble chart (Figure 5B). Then we found that the low-risk group had a higher immune score and ESTIMATE score, displaying a significant difference from the high-risk group (Figure 5C). In addition, the stronger correlation between immune function-associated biomarkers and ssGSEA scores showed that the low-risk group had a higher immune infiltration status (Figures 5D,E). The expression levels of immune checkpoints and/or their ligands may reflect the effect of immunotherapy. Then the correlation between the expression of 29 immune checkpoint genes and risk scores was investigated (Figure 5F). Low-risk patients exhibited a higher expression of 25 immune checkpoint genes, namely, BTLA, CD200, NRP1, LAIR1, CD244, LAG3, CD200R1, ICOS, CD40LG, CTLA4, CD48, CD28, HAVCR2, ADORA2A, KIR3DL1, TNFSF14, CD160, IDO2, IDO1, PDCD1, TNFRSF8, CD27, BTNL2, TIGIT, and CD86, while the remaining four were highly expressed in the high-risk group. Collectively, these results indicated that NRL signatures in the model affect tumor-infiltrating immune cell (TIC) infiltration and can be a candidate biomarker for immunotherapy.

### Clusters

**FIGURE 6:**
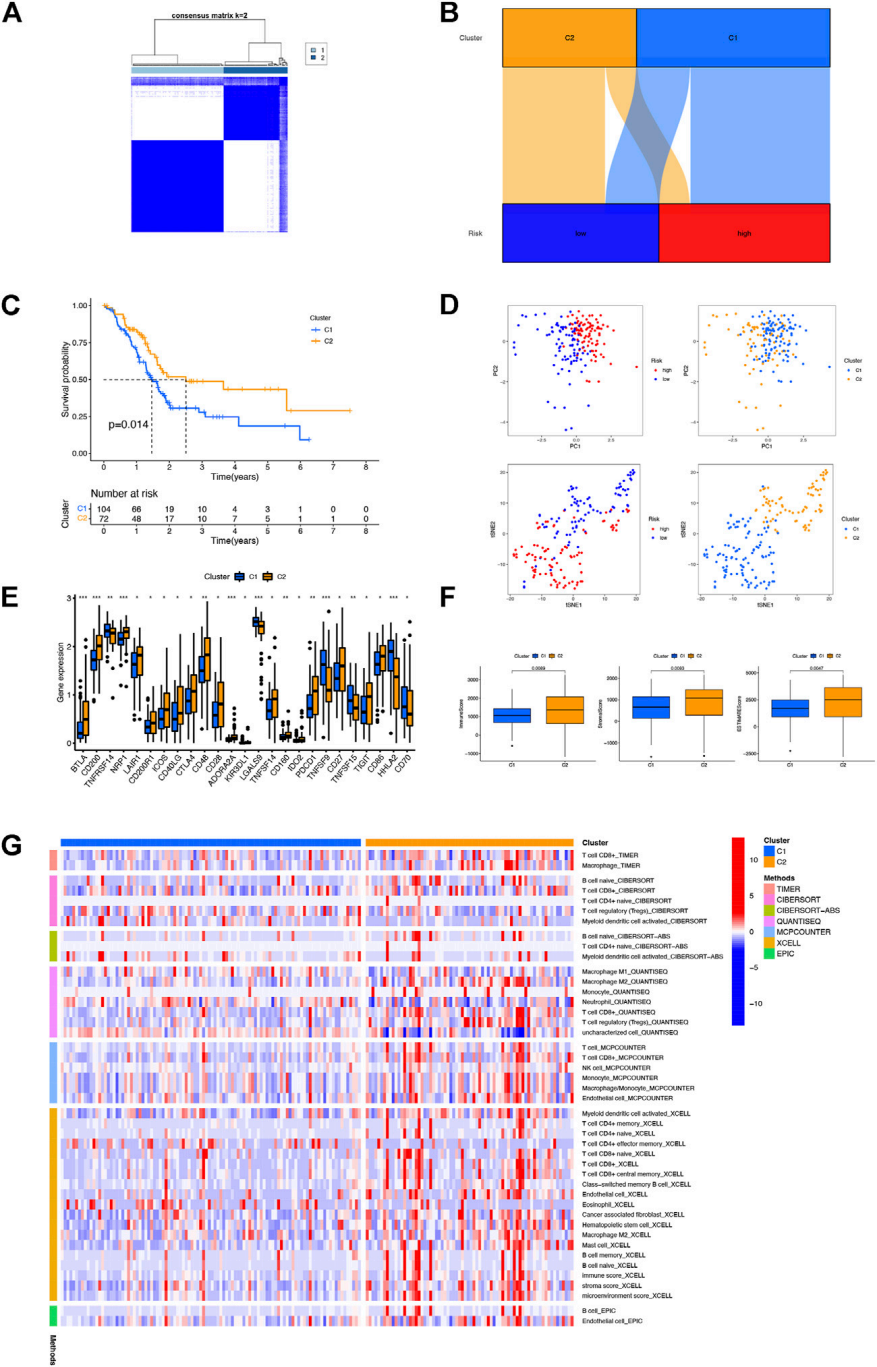
Distinction between different clusters. **(A)** Patients divided into two clusters by ConsensusClusterPlus. **(B)** Sankey diagram of different clusters and risk groups. **(C)** Kaplan–Meier survival curves of OS in clusters. **(D)** PCA and t-SNE of risk groups and clusters. **(E)** Differential expression of 25 checkpoint genes in different clusters. **(F)** Comparison of immune-related scores between clusters 1 and 2. **(G)** Heatmap of immune cells in clusters.

To evaluate the immune differences between different clusters, patients were divided into different clusters by the “ConsensusClusterPlus” R package based on the expression of NRLs in the model (Supplementary Figure S2). According to the results of clustering analysis, the optimal number of clusters was 2 (Figure 6A). The correlation between two clusters and risk groups is vividly shown in Figure 6B by a Sankey diagram. T-SNE and PCA indicated that the risk groups and two clusters could be distinguished clearly (Figure 6D). Furthermore, cluster 2 (C2) had better OS than cluster 1 (C1) (Figure 6C). As shown in Figures 6F,G, C2 had a higher immune score, estimate score, and immune cell infiltration, signifying a higher immune infiltration status than C1. With the immune checkpoint gene comparison, we found that C2 tends to have a higher expression of 19 immune checkpoint genes, namely, BTLA, CD200, NRP1, LAIR1, CD200R1, ICOS, CD40LG, CTLA4, CD48, CD28, ADORA2A, KIR3DL1, TNFSF14, CD160, IDO2, PDCD1, CD27, TIGIT, and CD86, while the remaining six immune checkpoint genes (TNFRSF14, LGALS9, TNFSF9, TNFSF15, HHLA2, and CD70) were highly expressed in C1 (Figure 6E). Based on the NRLs of different clusters, we could further explore the correlation between immunotherapy and NRL signatures in PAAD patients.

### Validation of the expression and prognostic value of linc01089 in PAAD

**FIGURE 7:**
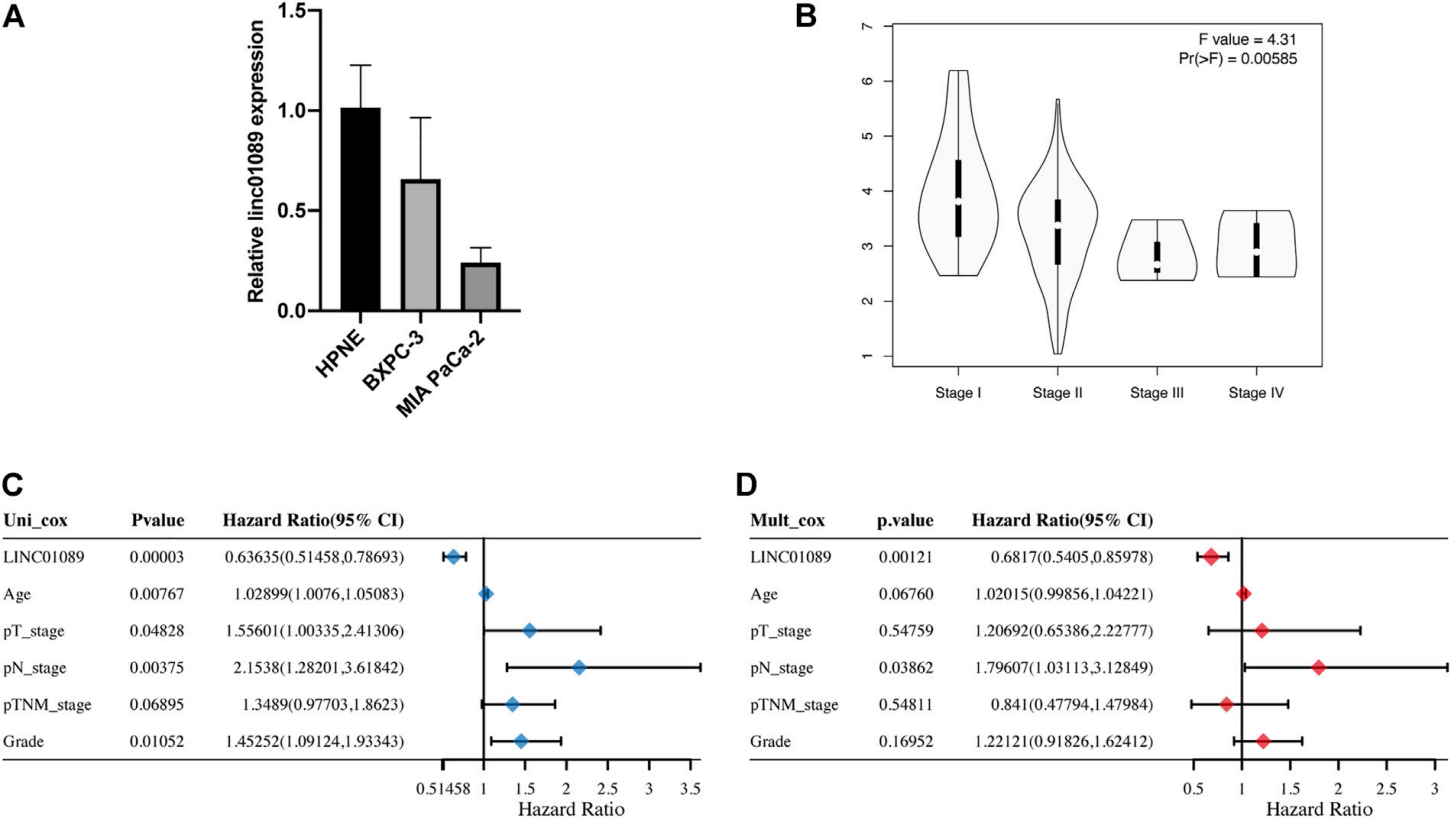
Validation of the expression and clinical characteristics of linc01089 in PAAD. **(A)** Expression of linc01089 was downregulated in PAAD cells than that in HPNE. **(B)** Correlation between linc01089 and the pathology stage in PAAD. **(C,D)** Univariate and multivariate analyses indicated that linc01089 and N stage were independent prognostic factors affecting the OS of PAAD patients.

The expression and prognosis value of linc01089 was explored based on RT-qPCR and TCGA dataset. Consistent with the previous results (Figure 1C), the expression of linc01089 was lower in PAAD cells than in HPNE (Figure 7A). Furthermore, the expression of linc01089 in stages III and IIV was lower than that in stages I and II (Figure 7B). Univariate and multivariate analyses revealed that the N stage and linc01089 expression were independent factors affecting the prognosis of PAAD patients (Figures 7C,D).

## Discussion

Pancreatic cancer is a highly malignant tumor of the digestive system. Because of its insidious symptoms and rapid disease progression, most patients present with locally advanced or metastatic disease at diagnosis and the treatment effect is not ideal (Park et al., 2021). Currently, the gene signatures of distinct molecular subtypes based on cell activities including autophagy (Hu et al., 2020), metabolic (Yang et al., 2020), immune (Kandimalla et al., 2020), and so on, show good predictive value for individual risk assessment in cancers. Given this reason, constructing a molecular prediction model in PAAD for guiding personalized therapy and predicting the prognosis is particularly important.

Necroptosis is a form of cell death which occurs downstream of protein kinase C-related kinase 1 and 3 (PRK1 and RIPK3) as members of the oligomeric complex termed the necrosome (Galluzzi et al., 2012). Accumulating studies illustrated that necroptosis plays a tumor inhibitory role (Newton, 2015) and reduces the incidence of metastasis by the accumulation of high ROS levels (Lawlor et al., 2015). LncRNAs involved in necroptosis have been continuously identified (Jiang et al., 2021). Harari-Steinfeld et al. (2021) found that lncRNA H19-derived microRNA-675 promotes liver necroptosis by targeting FADD. Wang et al. (2020) found that ammonia regulates chicken tracheal cell necroptosis via the lncRNA-107053293/miR-148a-3p/FAF1 axis. Although several NRL signatures of cancer have been reported (Wang and Liu, 2021; Zhao et al., 2021), there is no study associated with NRL signatures of pancreatic cancer. In this study, we established a prognostic model with five NRLs (U62317.1, AC099850.3, LINC01089, LINC01133, and LINC00996). On the basis of univariate and multivariate Cox regression analyses, the NRL signatures in the model are independent prognostic indicators, implying a moderate predictive value. In addition, the ROC curve and nomogram displayed a good predictive effect to predict prognosis and guide immunotherapy.

Among these NRLs in the model, all were associated with tumor progression in previous research. U62317.1 is identified as an lncRNA biomarker for the early diagnosis of oral squamous cell carcinoma (Li Y. et al., 2020). AC099850.3 is a member of the biomarkers that participate in autophagy, immune, and stemness (Jia et al., 2020; Wu et al., 2021; Zhang et al., 2021). LINC01089 inhibits the progression of gastric cancer (Guo and Li, 2020), cervical cancer (Li et al., 2021), colorectal cancer (Li and Guo, 2020), and lung cancer (Li X. et al., 2020) by sponging miRNA. LINC01133 aggravates the epithelial-mesenchymal transition through the Wnt/β-catenin pathway by silencing AXIN2 (Liu Y. et al., 2021). LINC00996 is a potential therapeutic target in pulmonary adenocarcinoma and squamous cell carcinoma (Yan et al., 2021). However, studies on cell activities and NRLs are still lacking. Thus, more experimental evidence of correlations between NRLs and PAAD is needed.

Growing evidence suggests that immune cells contribute to tumor progression when present in the tumor microenvironment (TME) (Hinshaw and Shevde, 2019). The efficacy of immunotherapy and overall survival were significantly correlated with the composition of the TME (Petitprez et al., 2020). In this study, patients with a low-risk score had a higher infiltration level with immune cells including B cells, CD8^+^T cells, dendritic cells, and NK cells and a stronger correlation with immune function, confirming the function of NRLs in tumor immune infiltration. Immune checkpoint blockade (ICB) enhances the efficiency of anti-tumoral immune response by the way of immune infiltration (Petitprez et al., 2020). Interestingly, CTLA4, IDO1, and PDCD1 were highly expressed in the low-risk group, implying that low-risk patients may benefit more from immunotherapy. There is a difference between different subtypes and immune scores which contribute to different prognoses and immunotherapy responses (DeBerardinis, 2020). Then we divided patients into two clusters on the basis of NRL expressions in the model. As expected, differences existed between clusters in the immune score and immune cell infiltration. C2 had a higher immune score and estimate score. At the same time, C2 had more immune cell infiltration and a higher expression of CTLA4 and PDCD1, which confirmed that C2 may benefit more from immunotherapy than C1. Above all, these NRLs could accurately predict the prognosis of PAAD patients and guide personalized immunotherapy.

There are still several limitations in the current study. First, external validation with data integrated with complete clinical and lncRNA information will be searched and analyzed subsequently. Second, functional experiments including cell and animal experiments will be in our plans to explore molecular mechanisms in-depth.

## Conclusion

All in all, we constructed a model with NRLs to predict prognosis and guide personalized immunotherapy in PAAD patients. Targeting these lncRNAs will be a promising way for systemic therapy failure and new pathways of immunotherapy. Therefore, molecular mechanisms between NRLs, necroptosis, and PAAD are worth investigating.

## Data Availability

PAAD RNA-seq dataset and Genotype-Tissue Expression project (GTEx) data used in this study are available *via* the UCSC Xena (http://xena.ucsc.edu/); further inquiries can be directed to the corresponding author.
