# Supplementary figures and images for: Integrated analysis of necroptosis-related lncRNAs for prognosis and immunotherapy of patients with pancreatic adenocarcinoma
Source: Front Genet. 2022 Aug 16;13:940794. doi: 10.3389/fgene.2022.940794 (PMC9424502; doi:10.3389/fgene.2022.940794)

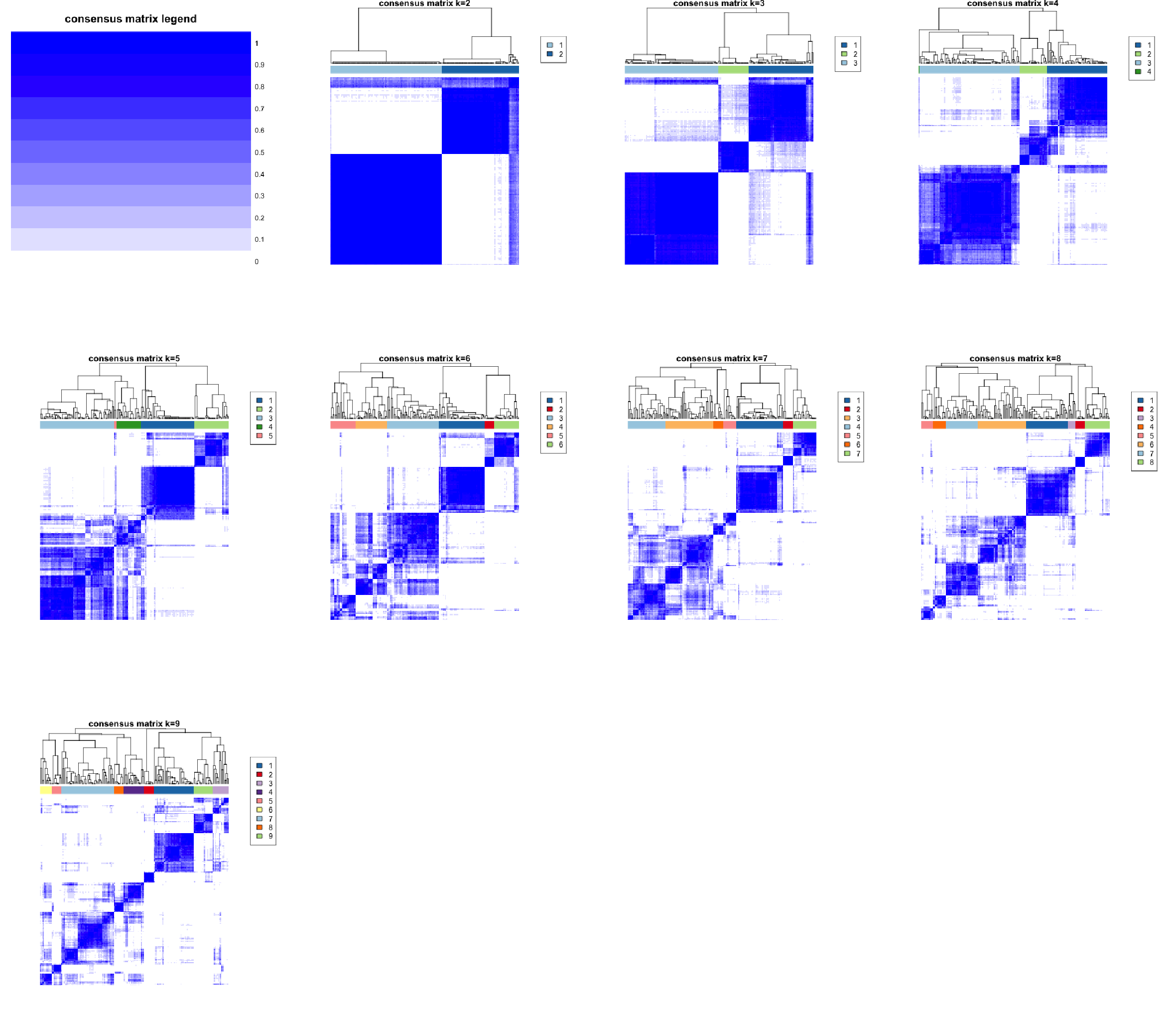

Supplement: Supplementary file 1 [file Image2.TIF]

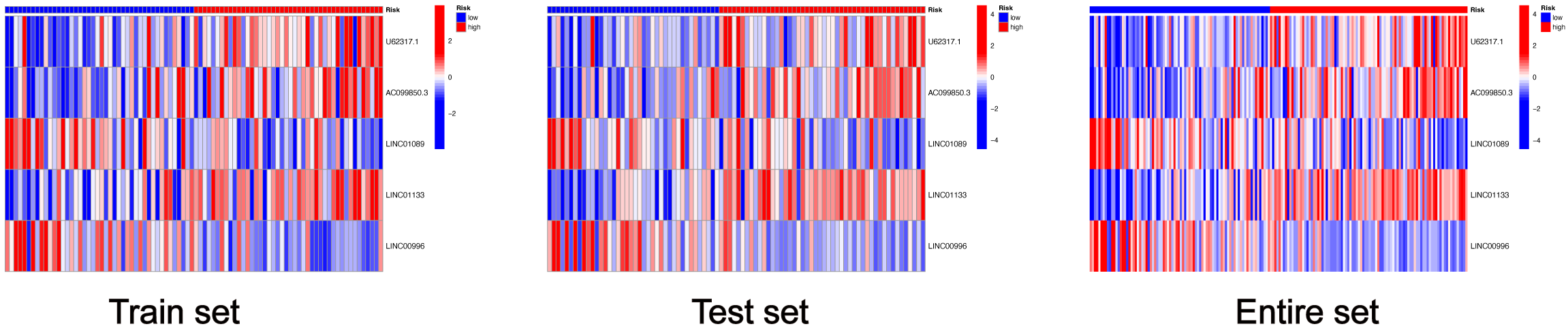

Supplement: Supplementary file 2 [file Image1.TIF]
